# Nonactivated and Activated Biochar Derived from Bananas as Alternative Cathode Catalyst in Microbial Fuel Cells

**DOI:** 10.1155/2014/832850

**Published:** 2014-08-26

**Authors:** Haoran Yuan, Lifang Deng, Yujie Qi, Noriyuki Kobayashi, Jiahuan Tang

**Affiliations:** ^1^Guangzhou Institute of Energy Conversion, Chinese Academy of Sciences, Guangzhou 510640, China; ^2^Key Laboratory of Renewable Energy, Chinese Academy of Sciences, Guangzhou 510640, China; ^3^Guangdong Institute of Eco-Environmental and Soil Sciences, 808 Tianyuan Road, Guangzhou, Guangdong 510650, China

## Abstract

Nonactivated and activated biochars have been successfully prepared by bananas at different thermotreatment temperatures. The activated biochar generated at 900°C (Biochar-act900) exhibited improved oxygen reduction reaction (ORR) and oxygen evolution reaction (OER) performances in alkaline media, in terms of the onset potential and generated current density. Rotating disk electron result shows that the average of 2.65 electrons per oxygen molecule was transferred during ORR of Biochar-act900. The highest power density of 528.2 mW/m^2^ and the maximum stable voltage of 0.47 V were obtained by employing Biochar-act900 as cathode catalyst, which is comparable to the Pt/C cathode. Owning to these advantages, it is expected that the banana-derived biochar cathode can find application in microbial fuel cell systems.

## 1. Introduction

Microbial fuel cells (MFCs) are an emerging green technology that employs the catalytic activity of microorganisms to degrade a wide range of organic matter and simultaneously generate electricity [1, 2]. The low activity of cathodic oxygen reduction reaction (ORR) is one of the most crucial factors limiting the performance of air-cathode microbial fuel cells (MFCs) [3]. To facilitate the slow kinetics of oxygen reduction at the cathodes, Pt-based materials are known to be excellent candidates for ORR catalysis. However, the usage of Pt-based catalyst is limited by its high cost and potentially poor stability due to catalyst poisoning. Hence, great efforts have been made to explore noble metal-free catalysts; inexpensive and highly available materials such as Co [4, 5], Fe [6], and MnO_2_ [7] were employed as MFC cathode catalysts.

In addition, metal-free catalysts for the ORR have gained significant attention because they do not suffer from crossover effects, have long-term operational stability, and are relatively cost-effective. Recently, carbon nanotubes (CNTs) [8, 9], carbon power [10], biochar [11], and activated carbon [12, 13] have been reported as promising cathode catalysts for fuel cell applications due to their high chemical stability, good electric conductivity, and enhanced mass transport capability. The power densities achieved from these cathodes were comparable to that from Pt cathodes, and the high ORR activity was attributed to the doping of electron-rich nitrogen to the carbon materials. In nitrogen-containing carbon materials, it is believed that either pyridinic or pyrrol/pyridone type nitrogen is responsible for the enhanced ORR activity [14]. These nitrogen functional groups transform to more thermally stable structures during heat treatment [15]. Furthermore nitrogen is known to be able to create defects on carbon, which may then increase the edge plane exposure and thus enhance the catalytic activity [16].

In this study, we used bananas to prepare carbon materials with activation and nonactivation and systematically investigated their catalytic activities toward ORR by voltammetry in a defined system. Subsequently, the as-prepared biochar was further employed as MFC cathode catalyst. The biochar cathode showed comparable capability to that of Pt-based catalyst in an MFC.

## 2. Materials and Methods

### 2.1. Synthesis and Activation of Biochar Samples

#### 2.1.1. Synthesis of Biochar

The biochars were prepared by the carbonization of the hydrothermal product of bananas (purchased from common supermarket) which was mentioned elsewhere [17]. In a typical procedure, 5 g of banana and 40 mL deionized water were placed in a commercial Teflon-lined autoclave with a capacity of 45 mL and then stirred by a glass rod. The autoclave was sealed and heated at 180°C for 12 h. The resulting hydrothermal carbonaceous solid was recovered by filtration, washed with ethanol and deionized water for several times, and then dried in an oven at 60°C, which was denoted as biochar. Subsequently, the biochar material was thermo-treated at 550°C or 900°C for 2 h under argon flow. After that, the samples were thoroughly washed by ethanol and deionized water and then dried in an oven at 100°C for 12 h. The samples are referred to as Biochar-550 and Biochar-900, respectively.

#### 2.1.2. Chemical Activation of Biochar

The activation methods of biochar samples were constructed as previously proposed by Dehkhoda et al. [18]. Briefly, the dried biochar powder samples were activated by 7 mol/L KOH solution and the mass ratio of pure KOH to biochar was 3.55. Then, the dried KOH-treated samples were ground to powder and placed in a tube furnace (Thermo Scientific Inc.) under nitrogen flow (258 mL/min). The system was initially heated to 300°C for 1 h and heated to 675°C for 2 h (dwell time) afterward. After washing with distilled water to neutral, the samples were mixed with 250 mL of 0.1 mol/L HCl. The activated biochar generated at 550°C and 900°C (different heat temperatures) are referred to as Biochar-act550 and Biochar-act900, respectively.

### 2.2. Characterization

The morphology of biochar samples was characterized with field emission scanning electronic microscopy (FESEM) (HITACHI, S-4800) with a field emission gun capable of 1~2 nm resolution. The samples did not need special pretreatment and were observed with SEM at 2.0 kV. The elements of biochar were analyzed by elemental-analyzer (Germany elementar Instrument Company, vario EL cube) using thermal conductivity detector.

The specific surface areas were measured by the Brunauer-Emmett-Teller (BET) method, in which N_2_ adsorption was applied at 77 K and Carlo Erba Sorptometer was used. X-ray power diffraction (XRD, X'Pert-PRO, PANalytical, Netherlands) analysis was performed with a Cu Kα target (*λ* = 0.154056 nm) radiation source.

### 2.3. Electrochemical Measurement

Cyclic voltammetric (CV) measurements were performed with an Autolab potentiostat (model PGSTAT 30) with a three-electrode (Ecochemie, The Netherlands). A Pt wire and a saturated calomel electrode (SCE) were used as the counter and reference electrodes, respectively. The catalysts coated glassy carbon (GC, 5.0 mm diameter) electrodes were used as working electrodes. CV measurements were performed from −0.6 V to 0.2 V at a scan rate of 100 mV/S in a 0.l mol/L KOH electrolyte. The electrolyte solution was bubbled with O_2_ to establish aerobic environment for 30 min prior to each scan series and 3 min between every two scans.

A rotating disc electrode (RED) half-cell setup was used to investigate the ORR. Electrochemical activity of the sample was studied using linear sweeping voltammetry (LSV) at a scan rate of 100 mV/S in 0.l mol/L KOH electrolyte. The working electrode was fabricated by casting Nafion-impregnated catalyst ink onto a glass carbon disk electrode (5 mm in diameter). A platinum foil and SCE were used as the counter and reference electrodes, respectively. Ultrahigh O_2_ was used for the purging of electrolyte. Catalyst activity toward the ORR was evaluated in oxygen-saturated electrolyte solution from 0.2 V to −0.8 V. The rotation rate is 500–2000 rpm. The catalytic performance for oxygen evolution reaction (OER) catalytic activity was also studied using LSV at a scan rate of 100 mV/S in a 0.l mol/LKOH or 0.25 mol/L solution from 1.0 V to −0.2 V versus SCE. For comparison purpose, commercial Pt/C (30 wt% platinum on carbon) was tested using the same procedure.

The preparation method of the working electrodes is as follows. In brief, 5 mg of catalyst was dispersed in l mL of 3 : 1 v/v water/ethanol mixed solvent with 5 *μ*L of Nafion solution (5 wt% Sigma-Aldrich). The mixture was then ultrasonicated for about 15 minutes to generate a homogeneous ink. Next, 8 *μ*L of the dispersion was transferred onto the glassy carbon disk, leading to a catalyst loading of ~0.2 mg/cm^2^. Finally, the as-prepared catalyst film was dried at room temperature.

### 2.4. MFC Configuration and Operation

Air-cathode single chamber MFCs with an inner volume of 12 mL were constructed as reported previously [19]. A cylindrical MFC chamber with a length of 1.7 cm and a diameter of 3.0 cm was made of Plexiglas. Both anode and cathode surface areas were 7 cm^2^. A nonwet proof carbon cloth and 30% wet proof carbon cloth were used for anode and cathode, respectively. The anode and cathode were placed on opposite sides with the oxygen catalyst coating layer facing the anode. The catalytic layer was prepared as described previously [4]. Briefly, the catalyst slurry was prepared by mixing 1 mg synthesized catalyst with 1 *μ*L water, and then Nafion solution (5% wt Sigma-Aldrich, 2 *μ*L) and ethanol (2 *μ*L) were added to the slurry and ultrasonicated for about 15 minutes to prepare a homogenous catalyst ink mixture. The obtained slurry was painted on one side of air cathode by using a brush and dried overnight at room temperature.

MFC anolyte culture media were 1 g/L sodium acetate solution. The medium solution contained NaH_2_PO_4_·2H_2_O (2.77 g/L), Na_2_HPO_4_·12H_2_O (11.40 g/L), NH_4_Cl (0.31 g/L), KCl (0.13 g/L), a vitamin stock solution (12.5 mL/L), and a mineral stock solution (12.5 mL/L).

After stable voltage outputs were achieved, power density curves were obtained by changing the circuit resistor from 10000 Ω to 50 Ω, and individual electrode potentials were measured versus saturated calomel electrode. All tests were conducted in batch mode in a 30°C incubator. The power was normalized by the projected surface area of the anode. All tests were conducted in triplicate, and the mean values are presented here.

## 3. Results and Discussion

### 3.1. Catalytic Activity of Banana Biochar toward ORR and OER

**Figure 1:**
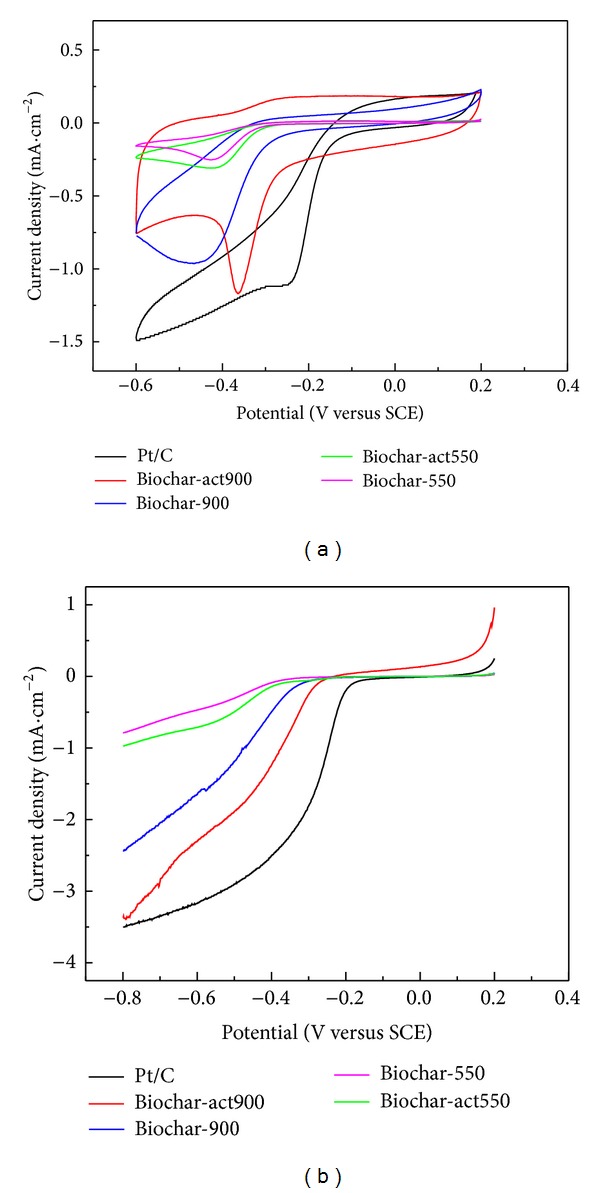
CV (a) and LSV (b) of various electrodes for the oxygen reduction at scan rate of 100 mV/S.

The electrochemical performance of the as-prepared banana biochar materials was tested by cyclic voltammograms at scan rate of 100 mV/s in a 0.l mol/L KOH electrolyte under aerobic (bubbled with O_2_) environment.  Figure 1(a) shows that as the thermotreatment temperature increases from 550°C to 900°C, all of the obtained nonactivated biochars (Biochar-550 and Biochar-900) presented a poor catalytic performance and just conductivity got improvement. However, after chemical activation, the catalytic performance of biochar which was obtained at the temperature of 900°C (Biochar-act900) got a great enhancement and at −0.36 V appeared an obvious oxygen reduction peak. When comparing with Pt/C (−0.21 V), though it has a more negative peak position, it got a higher catalytic current density. In addition, the banana biochar was widely available and inexpensive, so it can be a potential alternative to Pt/C in MFCs.

Half-cell testing was employed by LSV with a rotating disk electrode (RDE) to evaluate the ORR activities of catalysts (Figure 1(b)). Comparison of ORR activity was made with commercial Pt/C. As shown in Figure 1(b), the onset potential for Biochar-act900 was detected at −0.28 V, whereas they were −0.42 V, −0.41 V, −0.39, and −0.18 V for Biochar-550, Biochar-act550, Biochar-900, and Pt/C, respectively. At −0.8 V, Biochar-550, Biochar-act550, Biochar-900, Biochar-act900, and Pt/C afforded an ORR current density of −0.79 mA/cm^2^, −0.98 mA/cm^2^, −2.44 mA/cm^2^, −3.37 mA/cm^2^, and −3.50 mA/cm^2^, respectively. The current density of sample Biochar-act900 is higher than that of other samples, and the onset potential of Biochar-act900 is more positive than samples by 140 mV, 130 mV, and 110 mV, respectively. Biochar-act900 obtained relatively high adsorption of O_2_ and ORR performance and thus was expected to constitute a more effective cathode catalyst material than the other samples for MFCs, which is consistent with the above CV evaluations.

**Figure 2:**
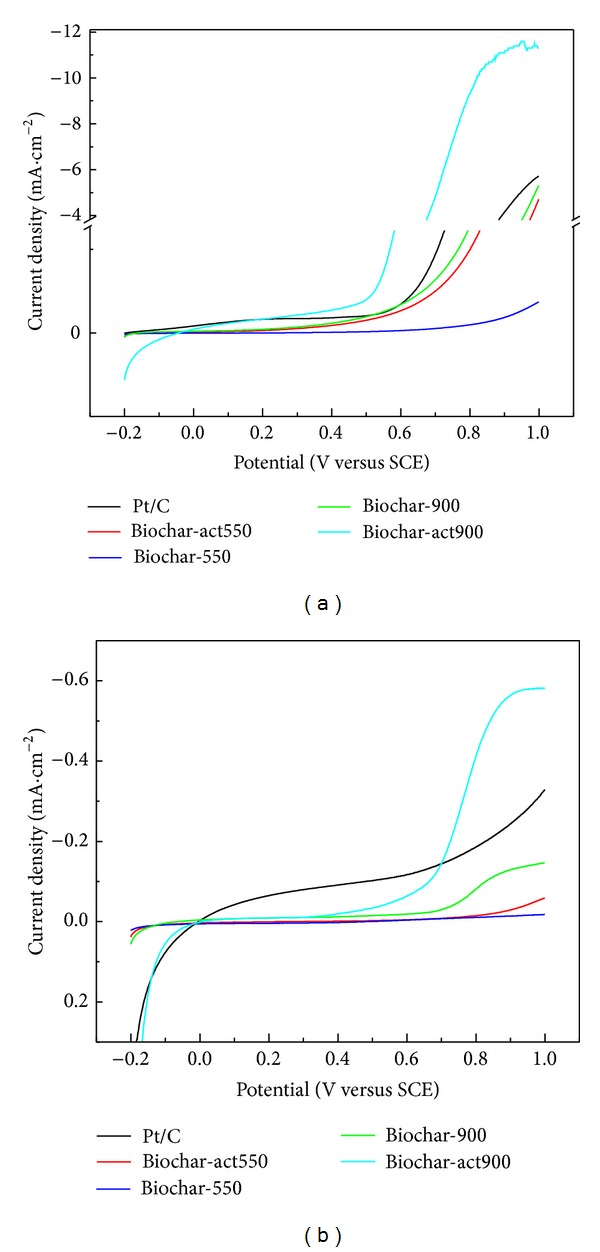
OER voltammetric curves in 0.1 mol/L KOH solution (a) and 0.25 mol/L K_2_SO_4_ solution (b).

Apart from the ORR activity, excellent OER activity is particularly critical for bifunctional catalysts [20]. The samples were then investigated as a catalyst for water oxidation under both alkaline and neutral conditions using electrochemical techniques (Figure 2). In the alkaline solution, LSV results (Figure 2(a)) show a distinct trend: the onset potential of Biochar-act900 is 0.42 V, 220 mV lower than that of Pt/C (0.64 V), 160 mV lower than that of Biochar-900 and Biochar-act550 (0.68 V), and 330 mV lower than that of Biochar-550 (0.85 V). In addition, Biochar-act900 produces a current density of 11.5 mA/cm^2^ at 1.0 V, 1.0 times higher than that of Pt/C (5.72 mA/cm^2^), 1.2 times higher than that of Biochar-900 (5.31 mA/cm^2^), 1.4 times higher than that of Biochar-act550 (4.71 mA/cm^2^), and 30.1 times higher than that of Biochar-550 (0.37 mA/cm^2^). Under the neutral condition (Figure 2(b)), the onset potential of Biochar-act900 is 0.52 V, 520 mV higher than that of Pt/C (0.0 V), 200 mV lower than that of Biochar-900 (0.72 V), and 340 mV lower than that of Biochar-550 and Biochar-act550 (0.89 V). Furthermore, the current density of Biochar-act900 at 1.0 V is 0.58 mA/cm^2^, 0.76 times higher than that of Pt/C (0.33 mA/cm^2^), 2.87 times higher than that of Biochar-900 (0.15 mA/cm^2^), 8.67 times higher than that of Biochar-act550 (0.06 mA/cm^2^), and 28.0 times higher than that of Biochar-550 (0.02 mA/cm^2^). That is, under alkaline solution, all of the samples were positive compared to in neutral solution. Among the four samples, Biochar-act900 has the highest activity, which is comparable to Pt/C.

**Figure 3:**
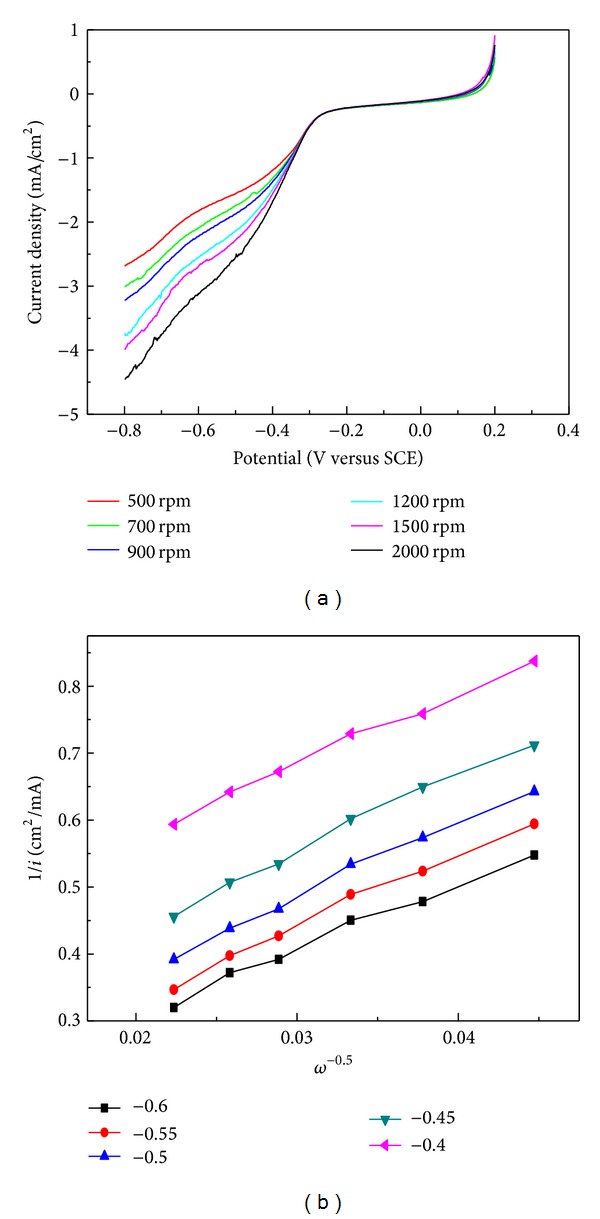
LSV plots at different rotation rates and corresponding Koutecky-Levich plots of sample Biochar-act900 at a scan rate of 100 mv/s.

The technique of rotating disk electrode (RDE) is also employed to investigate the kinetics of ORR. The RDE technique is beneficial in eliminating the effect of mass transport and thus can be used to more precisely evaluate the kinetics of ORR [21].  Figure 3 shows the RDE measurements at different rotating rates. The numbers of electrons transfer during ORR are calculated for Biochar-act900 using the Koutecky-Levich (K-L) equations [22]. Consider

(1) $$\begin{array}{c} \frac{1}{i}=\frac{1}{i_{k}}+\frac{1}{i_{l}},\\ i_{k}=nFkC_{0},\\ i_{l}={0.62nFD}_{0}^{2/3}\nu^{-1/6}C_{0}\omega^{1/2}, \end{array}$$

where *i*_*k*_ and *i*_*l*_ represent kinetic and diffusion-limiting current density (A/m^2^), respectively. And *n* is the electron number involved in ORR, *ω* is the rotation rate, *F* is the Faraday constant (96485 C/mol), *D*_0_ is the diffusion coefficient (1.86 × 10^−5 ^cm^2^/s in 0.1 mol/L KOH), *ν* is the kinematic viscosity of the electrolyte (0.01008 cm^2^/s in 0.1 mol/L KOH), *C*_0_ is the saturation concentration of O_2_ in the electrolyte (1.2 mmol/L in 0.1 mmol/L KOH), and *k* is the electron-transfer rate constant. By linearly fitting the K-L plots of *i* ^−1^ versus *ω* ^−0.5^, the *n* values for ORR can be obtained, quantitatively. Combining Koutecky-Levich (K-L) equations and using the slopes from Figure 3(b), the number of electrons transferred, which gives 2.89, 2.62, 2.55, 2.49, and 2.71, 2.65 in average, which is relative to the number of transferred electrons for Pt/C which is assumed to be 4.0.

### 3.2. Morphological and Structural Characteristics of Biochar

**Figure 4:**
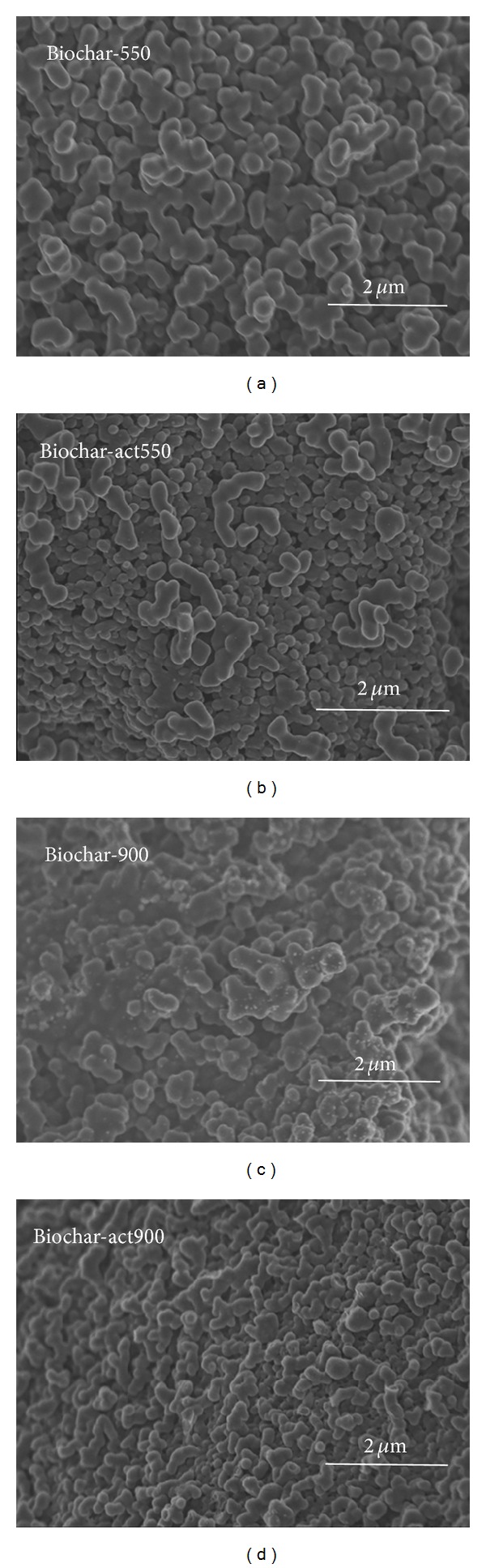
SEM images of the biochar at different hydrothermal carbonization and after chemical activation.

FE-SEM images illustrate the images of banana after hydrothermal carbonization and thermotreatment (Figures 4(a) and 4(c)) and subsequent chemical activation (Figures 4(b) and 4(d)). As can be seen in the figures, the shape and size of the obtained carbon materials at different temperatures are similar, but the size becomes smaller after chemical activation. However, the specific areas are significantly different (Table 1). Biochar-900 exhibits a larger specific surface area than Biochar-550, indicating that the thermotreatment temperature is positive for the preparation of carbon material with larger specific area; this result is different from that which was reported by Wang et al. [17]. And after chemical activation, the specific surface area increases. For example, the specific surface area of Biochar-act900, about 28.0 m^2^/g larger than Biochar-900, and Biochar-act550, about 11.5 m^2^/g larger than Biochar-550 (Table 1). The observations are consistent with the above electrochemical evaluations.

**Figure 5:**
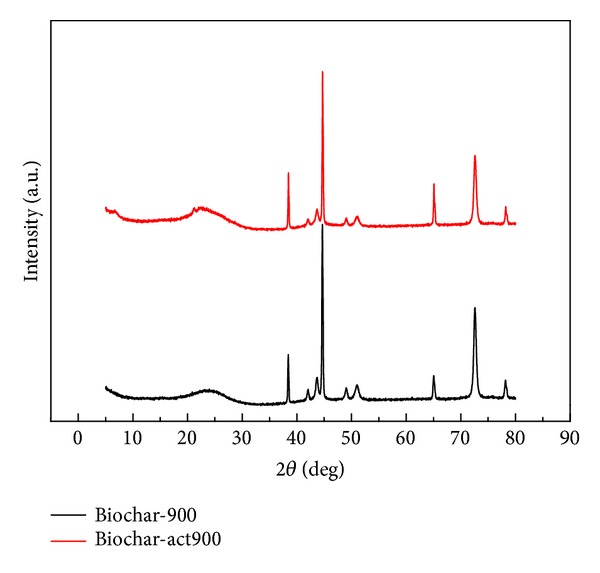
XRD patterns of Biochar-900 and Biochar-act900.

Figure 5 shows the XRD pattern of Biochar-900 and Biochar-act900, and both of them presented broad or sharp peaks at 2*θ* of about 23°, 38°, 43°, 45°, 49°, 51°, 65°, 73°, and 79°, which are probably indexed as an orthorhombic unit cell having three lattice parameters *a* = 4.12 Å, *b* = 4.54 Å, and *c* = 4.14 Å. The results reveal that chemical activation has no significant influence on the crystal form of the as-prepared biochar.

### 3.3. Application to Microbial Fuel Cell

**Figure 6:**
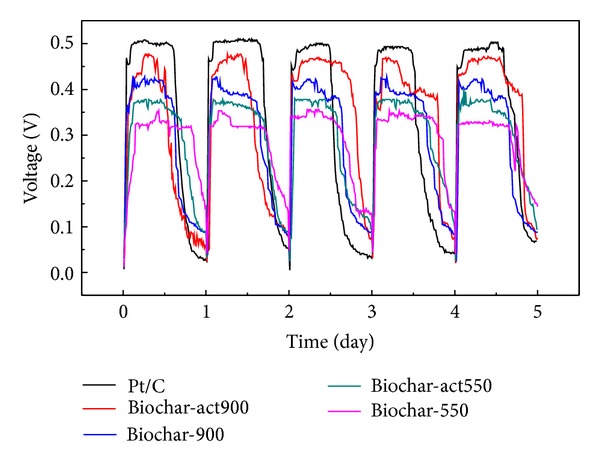
Voltage output at 1 kΩ loading achieved in consecutive electricity generation cycles.

**Figure 7:**
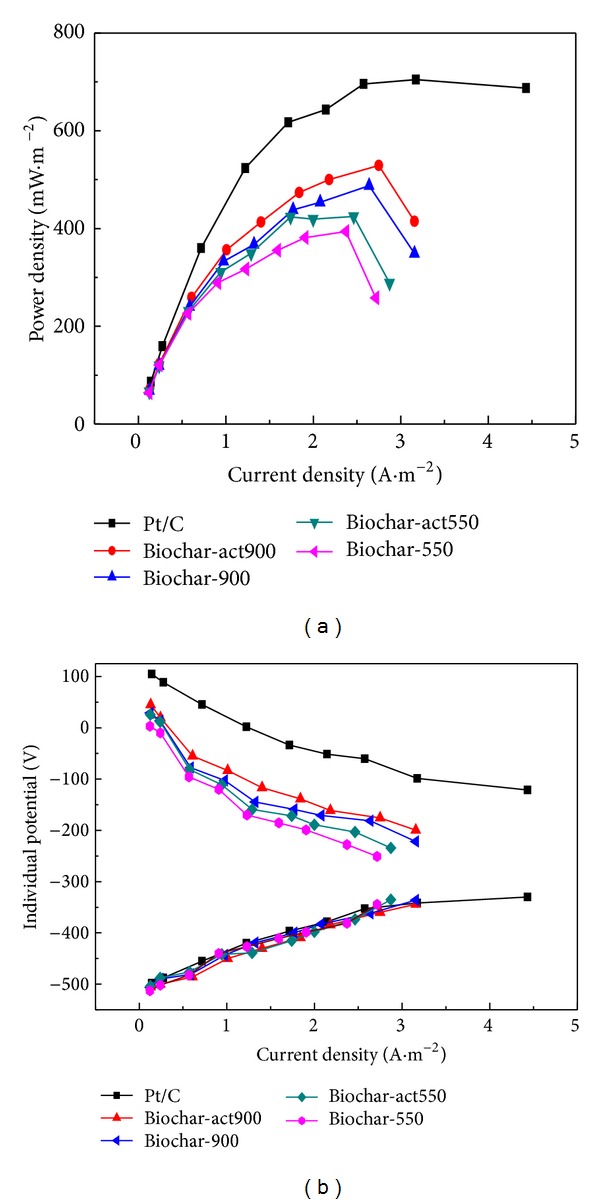
Performance of MFCs equipped with biochar. (a) Polarization curves of the MFCs with various cathode catalysts, (b) individual potentials versus current density curves.

Hydrothermal-based synthesis and thermotreatment of carbon material from banana was used in supercapacitor [17]. However, their application in MFCs and further comparative analysis of their performance are rarely reported. In this section, we investigated the catalytic properties of biochar as cathode catalyst in MFCs. The performance of MFCs with Biochar-550, Biochar-act550, Biochar-900, and Biochar-act900 as cathode catalysts was assessed by monitoring cell output, anode and cathode polarization, and power density (Figures 6 and 7). As shown in Figure 6, the MFC with Biochar-act900 as cathode catalyst presents a maximum stable voltage of 0.47 V, about 0.03 V lower than that with Pt/C (about 0.5 V), and 0.05 V, 0.10 V, and 0.14 V higher than that with Biochar-900 (about 0.42 V), Biochar-act550 (about 0.37 V), and Biochar-550 (about 0.33 V) as cathode catalyst, respectively.

Power densities and polarization curves were studied by using polarization curve for different catalysts loaded air cathodes as shown in Figure 7(a). The MFC with Biochar-act900 as cathode catalyst produced a power density of 528.2 mW/m^2^, which was lower than that of Pt/C catalyst (695 mW/m^2^). However, power densities with other catalysts were 483.7 mW/m^2^, 424.6 mW/m^2^, and 393.7 mW/m^2^ for Biochar-900, Biochar-act550, and Biochar-550, respectively, all lower than the power density produced by the MFC with Biochar-act900. The results suggested that the Biochar-act900 was feasible cathode catalyst for MFC compared to Pt/C. All the results were compared with the same amount of catalyst loading on the air cathode and all other conditions were the same.  Figure 7(b) showed the curves of individual electrode potentials versus current densities. It can be observed that the potential variations for cathode were much more distinctive compared with the anode potential variation for various MFCs. Anode potentials were almost the same for different MFCs whereas cathode potentials varied in a wide difference. The variation in cathode potentials was mainly due to the efficiency of different catalysts towards oxygen reduction.

Gouérec et al. [23] pointed out that nitrogen functionality has positive importance on the ORR activity. The elements of biochars at different treatments were detected by the element analyzer, and the result was present in Table 2. As shown in Table 2, sample Biochar-act900 contains the highest nitrogen content about 3.57%, followed by Biochar-900 (3.34%), Biochar-act550 (2.86%), and Biochar-550 (2.81%). So the higher power density, maximum stable voltage, and ORR activity may be attributed to the existence of nitrogen element and large specific surface area. But the specific reasons and mechanism of action need to be further studied.

## 4. Conclusions

In this study, biochars derived from banana at different thermotreatment temperatures and with or without chemical activation were used as cathode catalysts in MFCs. Our experimental results demonstrate that the thermotreatment temperatures and chemical activation play an active role for the preparation of high active cathode catalyst. Electrochemical analysis showed that the Biochar-act900 had very high catalytic activity for the ORR. Morphologic and element analyzer revealed that high specific surface area and enriched nitrogen contents in the Biochar-act900 might have jointly contributed to the high catalytic activity for the ORR. With the proposed cathode catalyst, a maximum power density of 528.2 mW/m^2^ and with the maximum stable voltage of 0.47 V was obtained, which was comparable to the Pt/C cathode catalyst. The results demonstrated that biochar derived from banana can be a potential alternative to Pt in MFCs.

## Figures and Tables

**Table 1:** The specific surface areas of biochars at different treatments.

Sample	Thermotreatment temperature (°C)	Chemical activation	Specific surface area (m^2^/g)
Biochar-550	550	No	105.1
Biochar-act550	550	Yes	116.6
Biochar-900	900	No	144.3
Biochar-act900	900	Yes	172.3

**Table 2:** The elements of biochars at different treatments.

Sample	Thermotreatment temperature (°C)	Chemical activation	C (%)	N (%)	H (%)
Biochar-550	550	NO	82.09	2.81	1.35
Biochar-900	550	NO	84.74	3.34	2.46
Biochar-act550	900	Yes	85.89	2.86	1.17
Biochar-act900	900	Yes	85.97	3.57	1.34
